# Presence of zoonotic black-pigmented periodontal pathogens in the oral microbiota of pet and stray cats

**DOI:** 10.17221/59/2022-VETMED

**Published:** 2023-02-20

**Authors:** Volkan Ozavci, Hafize Tugba Yuksel Dolgun, Sukru Kirkan

**Affiliations:** ^1^Department of Microbiology, Faculty of Veterinary Medicine, Dokuz Eylul University, Izmir, Türkiye; ^2^Department of Microbiology, Faculty of Veterinary Medicine, Aydin Adnan Menderes University, Aydin, Türkiye

**Keywords:** PCR, *Porphyromonas gingivalis*, *Porphyromonas gulae*, *Prevotella nigrescens*

## Abstract

Black-pigmented bacteria are one of the neglected species to cause periodontal disease in cats, and they are also zoonotic agents that pose an infection risk to humans. In this study, we aimed to determine the presence of *Porphyromonas gingivalis*, *Porphyromonas gulae* and *Prevotella nigrescens* in the oral microbiota of pet and stray cats. Dental swab samples were taken from 25 pet cats and 25 stray cats with symptoms of periodontal disease and then investigated by multiplex polymerase chain reaction using 16S rRNA species-specific primers. As a result of the multiplex PCR analysis, *P. gingivalis* 3/25 (12%), *P. nigrescens* 1/25 (4%), *P. gingivalis* + *P. gulae* 7/25 (28%), *P. gingivalis* + *P. nigrescens* 1/25 (4%), *P. gulae* + *P. nigrescens* 1/25 (4%), and *P. gingivalis* + *P. gulae* + *P. nigrescens* 2/25 (8%) were molecularly typed in the pet cats. In addition, 1/25 (4%) of *P. gulae* and 21/25 (84%) of *P. gingivalis* + *P. gulae* were typed in the stray cats. In 10/25 (40%) pet and 3/25 (12%) stray cat samples, no bacteria were detected by molecular typing. In summary, the results provide strong evidence that black-pigmented zoonotic pathogens are associated with cat periodontal disease.

Black-pigmented, asaccharolytic, non-motile, Gram-negative anaerobic bacteria species like *Porphyromonas* spp. and *Prevotella* spp. are present in plaque and periodontal gum pockets. Plaque can damage the gingival tissue and leads to growing pathogenic Gram-negative bacteria (*Porphyromonas* spp., *Prevotella* spp., etc.) below the gum line. The bacterial species can also be detected in sialadenitis, gingivitis, periodontal tissue inflammation, and endodontic infections in cats (Kolenbrander et al. 2010; Gilpin et al. 2017; Whyte et al. 2017; Shenbakam et al. 2021; Tett et al. 2021). Periodontal diseases have a prevalence of 70% and occur in cats of all ages. Although diet and some diseases affect its prevalence and severity, it is generally accepted that the disease progresses with age (O’Neill et al. 2014). In addition, some diseases such as diabetes, hyperthyroidism, hyperadrenocorticism, feline immunodeficiency virus (FIV), and feline leukaemia virus (FeLV) may increase the sensitivity against periodontitis disease in cats (Miceli et al. 2021). Some species may provide the basis for colonisationion and different disease factors. For example, mycotic agents can be detected in cats with *Porphyromonas* spp. bacteria in their oral microbiota (Whyte et al. 2017). *Porphyromonas gingivalis* (*P. gingivalis*) is known as a zoonotic dominant pathogen in the oral microbiota of cats and it can bind to salivary enzymes, extracellular matrix proteins, and commensal bacterial surfaces. Periodontal tissues have an immune system against bacterial invasion. However, due to the infection caused by periodontopathogenic bacteria, the natural and acquired immunity of the host may be impaired and destruction may occur in the bone tissue (Amano 2007; Adler et al. 2016; Usui et al. 2021). *Prevotella* spp. are pigmented or non-pigmented asaccharolytic bacteria that can live in the oral cavity and gastrointestinal tract of animals and humans (Tett et al. 2021). Mostly, 10–20% of bite wounds become infected, including 30–50% of cat bites. The infection rates are between 4–25% in the case of cat’s bite wounds (Rothe et al. 2015; Dimcic et al. 2020). It has also been reported that black-pigmented zoonotic agents can be detected from many local or systemic diseases in humans (Figure 1) (Cohen et al. 2003; Gomes et al. 2005; Hamada et al. 2008; Wegner et al. 2010; Fernandez-Canigia et al. 2015; Venkataraman and Almas 2015; Gilpin et al. 2017; Dominy et al. 2019; Acuna-Amador and Barloy-Hubler 2020; Tett et al. 2021; Nonaka et al. 2022). This study aims to determine that zoonotic pathogens, such as *P. gingivalis*, *P. gulae*, and *P. nigrescens*, show the potential to cause many diseases in humans, in the oral microbiota of pet cats cared for at home, and in cats at large. Samples were collected from pet cats, exposed to ordered veterinary controls, and fed with commercially formulated dry food, and from stray cats without health controls and randomly fed with mixed food (leftovers). The data obtained in this study will be a protective guide against possible diseases that may pose a risk for both veterinarians and animal lovers who keep cats at home and on the streets.

**Figure 1:**
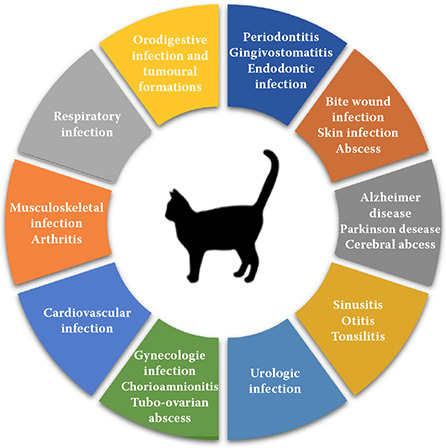
Some diseases in humans associated with cat-borne zoonotic *Porphyromonas* spp. and *Prevotella* spp. (Cohen et al. 2003; Gomes et al. 2005; Hamada et al. 2008; Wegner et al. 2010; Fernandez-Canigia et al. 2015; Venkataraman and Almas 2015; Gilpin et al. 2017; Dominy et al. 2019; Acuna-Amador and Barloy-Hubler 2020; Tett et al. 2021; Nonaka et al. 2022)

## MATERIAL AND METHODS

### Study design and sample collection

**Figure 2:**
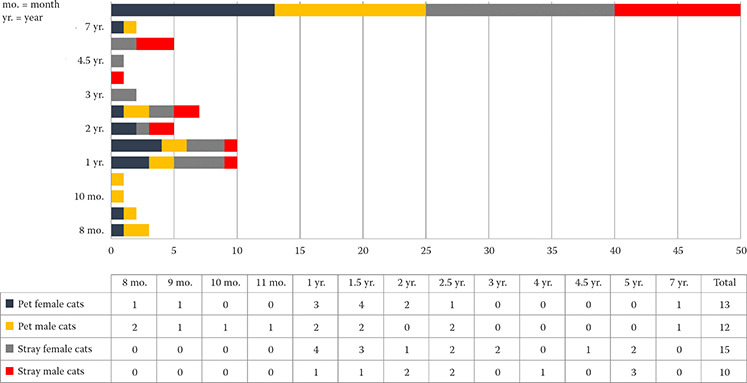
Distribution of the age and number of cats (pet-stray)

In this study, dental swab samples were collected from 25 pet cats (living at home) and 25 stray cats (living in urban conditions) examined in veterinary clinics in Izmir province and its districts between November 2021 and January 2022 with symptoms of periodontal disease. The pet cats were fed with professional dry food, and the stray cats were fed with mixed food (dry food, leftovers). Swabs were collected by rubbing swab sticks on the surface of the canines, gingival mucosa, dental plaque, and premolars. The samples were transferred to Aydin Adnan Menderes University Veterinary Faculty Microbiology Department with the Stuart transport medium under the cold chain system. Of the pet cats, 13 (52%) were grouped as female and 12 (48%) as male. The age range of cats was determined (by the pet owner and veterinary consultation) as follows; 20 (80%) adolescents (6–24 months), 3 (12%) adults (3–6 years), and 2 (8%) mature (7–10 years). Of the stray cats, 15 (60%) were grouped as female and 10 (40%) as male, 16 (64%) of them were adolescents, and 9 (36%) of them were adults (Figure 2). In the pet cats, 2 male cats were defined as infertile (neutered), and, in the stray cats, 5 male (neutered) and 10 female cats (spayed) were defined as infertile.

### DNA extraction from the swabs

The swab samples were transferred to Eppendorf tubes containing 500 microlitres (μl) of sterile distilled water in the laboratory. Then, they were vortexed and centrifuged at 7 826 × *g* for 6 minutes. The sediment at the bottom of the tubes was collected and dissolved in 100 μl of sterile 0.9% NaCl solution. Afterward, the bacterial DNA was extracted for each sample using an extraction kit (Thermo Fisher^®^, Waltham, MA, USA) designated by the manufacturer. The DNA quantity was measured with a Nanodrop device (Maestrogen^®^, Hsinchu City, Taiwan) and recorded. The obtained DNAs were stored in a deep freezer at –20 °C until used in the polymerase chain reaction (PCR) studies. In our study, *P. gingivalis* (ATCC 33277), *P. gulae* (ATCC 51700), and *P. nigrescens* (ATCC 33563) were used as the positive controls.

### The primers used and PCR analysis

A PCR analysis was applied by using 16S rRNA species-specific primers after extraction. The universal primers and *P. gingivalis*, *P. gulae*, and *P. nigrescens* species-specific primers were designed as stated by Ozavci et al. (2019). The 16S rRNA PCR reaction for the detection of total bacteria was examined at a total volume of 25 μl, including 2X *Taq* Mastermix (GenetBio^®^, Daejeon, Republic of Korea) 12.5 μl, 50 millimolar (mM) MgCl_2_ 0.75 μl, 50 μM forward and reverse universal primer for each 1 μl, 50–100 ng template DNA 5 μl, and completed with nuclease-free water. The PCR temperature cycling conditions were as follows: initial denaturation at 95 °C for 5 minutes (min), 1 min denaturation at 95 °C, 1 min annealing at 55 °C, 1 min elongation at 72 °C with 30 cycles, and final elongation at 72 °C for 10 min with 1 cycle (Rodrigues et al. 2019). Then, the species-specific multiplex PCR was performed on the positive samples with the universal primers. For the multiplex PCR reaction, a total volume of 25 μl was examined, including 2X *Taq* Mastermix (GenetBio^®^) 12.5 μl, 50 mM MgCl_2_ 0.75 μl, 50 μM *P. gingivalis*, *P. gulae* and *P. nigrescens* specific forward and reverse primer mix for 1 μl, 50–100 nanogram (ng) template DNA 5 μl and completed with nuclease-free water. Afterward, amplification was carried out under the following conditions: one cycle of 95 °C for 5 min for pre-denaturation, 94 °C for 30 seconds (s), 62 °C for 30 s, 72 °C with 30 cycles for 30 s, 72 °C for 5 min at a final elongation of 1 cycle. The PCR products were then electrophoresed at 80 V/cm for 40 min on a 2% agarose gel containing ethidium bromide. The agarose gel containing the products was scanned with a UV transilluminator system (Vilber Lourmat, Collégien, France) and evaluated based on the amplicon size length of the target genes (Takahashi and Yamada 2000; Sanai et al. 2002; Rodrigues et al. 2019).

## RESULTS

**Figure 3:**
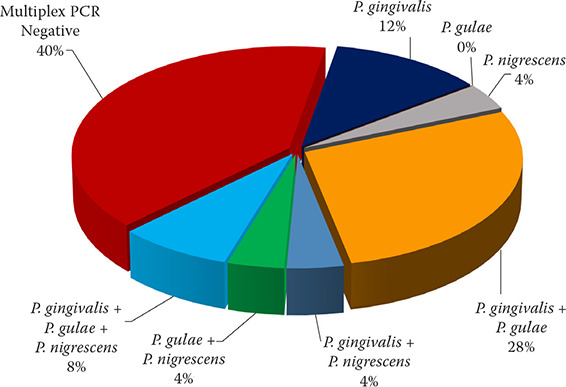
Multiplex PCR analysis results of the pet cats

**Figure 4:**
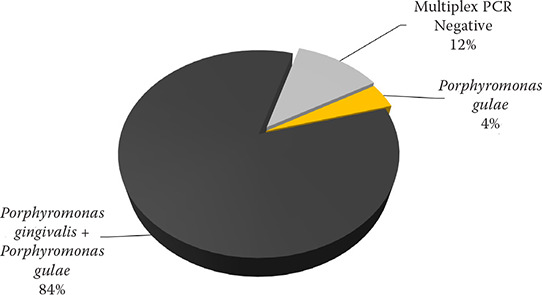
Multiplex PCR analysis results of the stray cats

**Figure 5:**
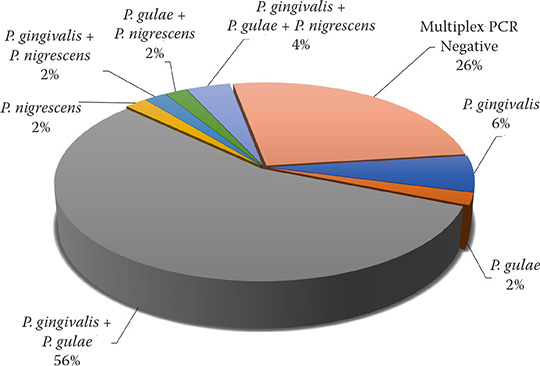
Distribution of the species detected from the pet and stray cats

**Figure 6:**
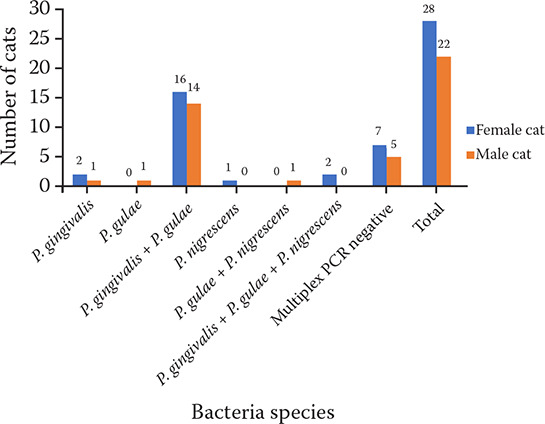
Distribution of the bacteria detected in the cats by gender

**Figure 7:**
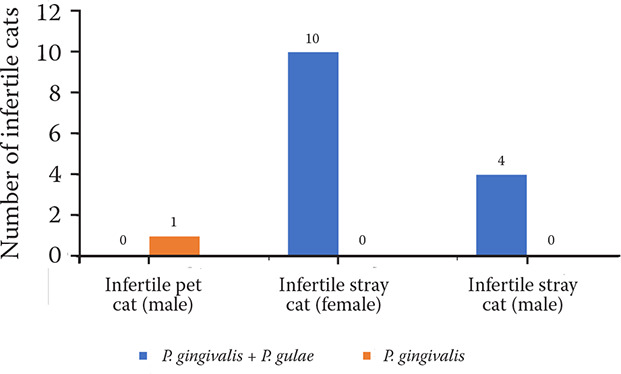
Distribution of the positive detected bacteria in the infertile cats

Multiplex PCR testing was performed on the positive samples obtained from the 16S rRNA PCR test with 50 samples. *P. gingivalis*, *P. gulae*, and *P. nigrescens* species-specific primers were used for the molecular typing. The findings were determined in the pets as follows: *P. gingivalis* (3/25, 12%), *P. nigrescens* (1/25, 4%), *P. gingivalis + P. gulae* (7/25, 28%), *P. gingivalis* *+ P. nigrescens* (1/25, 4%), *P. gulae* *+ P. nigrescens* (1/25, 4%), *P. gingivalis* *+ P. gulae* *+ P. nigrescens* (2/25, 8%). However, the PCR analysis was performed in 10/25 (40%) of the pet cat samples, but the bacterial existence was not identified (Figure 3). In the stray cats, *P. gulae* 1/25 (4%) and *P. gingivalis* + *P. gulae* 21/25 (84%) were determined in the molecular typing, but could not be detected in any of the results in 3/25 (12%) samples (Figure 4). In all the samples (50), *P. gingivalis* 6% (3/50)*, P. gulae* 2% (1/50)*, P. gingivalis* *+ P. gulae* 56% (28/50)*, P. nigrescens* 2% (1/50), *P. gingivalis* *+ P. nigrescens* 2% (1/50)*, P. gulae* *+ P. nigrescens* 2% (1/50)*,* and *P. gingivalis* *+ P. gulae + P. nigrescens *4% (2/50) were identified at the specified rates. However, the multiplex PCR negative samples were identified at a rate of 26% (13/50) (Figure 5). The presence of bacteria in cats according to gender is grouped as follows: *P. gingivalis* + *P. gulae* 60.71% (17/28) in the females and 59.09% (13/22) in the males, *P. gulae* 4.54% (1/22) in the males, *P. gingivalis* in the females 7.14% (2/28), *P. nigrescens* 3.57% (1/28) in the females, *P. gingivalis* + *P. gulae *+ *P. nigrescens* 7.14% (2/28) in the females and *P. gulae* + *P. nigrescens* 4.54% (1/22) in the males (Figure 6). *P. gingivalis* + *P. gulae* were identified 66.66% (10/15) from the females of the infertile stray cats. Conversely, *P. gingivalis* + *P. gulae* were identified at a rate of 40% (4/10) in the infertile stray male cats, and *P. gingivalis* was identified at a rate of 8.33% (1/12) in the infertile pet male cats (Figure 7). Among the infertile pet cats, *P. gingivalis* *+ P. gulae* were identified from only one 30-month-old male cat.

## DISCUSSION

Both *P. gingivalis* and *P. gulae* are anaerobic pathogens that cause changes in the oral microbiota composition associated with periodontitis and systemic diseases (Nomura et al. 2020). As a result of gingivitis, the subgingival biofilm, anaerobic Gram-negative secondary bacteria colonies, such as *Prevotella* spp. and *Porphyromonas* spp., can attach to the animals’ oral tissues (Golynska et al. 2017). Many studies have reported that more than 70% of cats are exposed to periodontal disease by at least 24 months of age (Rodrigues et al. 2019). This study investigated the presence of detected *P. gingivalis*, *P. gulae*, and *P. nigrescens* as black-pigmented Gram-negative anaerobic zoonotic pathogens in the oral microbiota of cats aged between 12 and 30 months. *P. gingivalis* and *P. gulae* were the most relevant pathogen in the periodontal disease in cats. *P. intermedia* and *P. gingivalis* have been known as pathogens associated with the development and progression of periodontitis (Sanai et al. 2002). In this study, oral bacterial diversity was found to be the higher rate in pet cats. The findings show that *P. gulae* can be detected with both *P. gingivalis* and *P. nigrescens* in a mixed infection in pet cats. Interestingly, *P. gulae* were identified as a single infective pathogen in the stray cats. Bacterial species, such as *Staphylococcus aureus*, *Streptococcus pyogenes*, *Corynebacterium* spp., *Listeria* spp., and *Pasteurella multocida*, are considered to be frequently isolated from stray cat bite wounds. However, our findings highlight that it would be beneficial to clinically evaluate the black-pigmented bacterial species in bite wounds. In addition, we detected a more common multi-bacterial distribution in pet cats according to the stray cats. It has been reported that the formulated dry food (commercial) consuming cats had more oral *Porphyromonas* spp. diversity than cats fed with canned wet food or fresh meat. Therefore, the cats fed in this way would be taken 12% more carbohydrates with the diet (Adler et al. 2016; Spears et al. 2017). Different diets (rich in fat and low in fibre) and lifestyles may reduce the growth of *Prevotella* spp. (Tett et al. 2021). Glucose is an essential factor for *Prevotella* spp. that promotes anaerobic growth and supports intracellular polysaccharide aggregation (Takahashi and Yamada 2000). In our findings showed that there were bacterial differences in the oral microbiota and signs of periodontal disease in domestic and stray cats fed with different diets. The isolation of *P. nigrescens* from pet cats fed with commercial food can be considered a physiological adaptation and further research on this subject may be useful to establish a relationship. However, neutering, the gender, and the age range were not considered as factors affecting the bacterial diversity in the mouth.

In conclusion, cats (pets or strays) can host black-pigmented pathogens, such as *P. gulae*, *P. nigrescens*, and *P. gingivalis,* in their dental plaque and oral microbiota. Nutrition may influence the development and composition of the oral microbiome in cats. For this reason, the effect of the diet on the bacterial diversity in the oral microbiota of cats should be investigated in more studies. A microbiological analysis of the gingival pockets using molecular-based tests in both veterinary and human clinical practice can provide the basis for increasing the efficacy of any potential treatment and prophylaxis. As a preventive approach, regular prophylactic clinical periodontal examinations of both pet and stray cats by veterinarians and informing their owners/feeders will be helpful in terms of preventing zoonotic infections.

## References

[R1] Acuna-Amador L, Barloy-Hubler F. Porphyromonas spp. have an extensive host range in ill and healthy individuals and an unexpected environmental distribution: A systematic review and meta-analysis. Anaerobe. 2020 Dec;66:102280. 33011277 10.1016/j.anaerobe.2020.102280

[R2] Adler CJ, Malik R, Browne GV, Norris JM. Diet may influence the oral microbiome composition in cats. Microbiome. 2016 Jun 9;4(1):1-9. 27277498 10.1186/s40168-016-0169-y PMC4899902

[R3] Amano A. Disruption of epithelial barrier and impairment of cellular function by Porphyromonas gingivalis. Front Biosci. 2007 May 1;12:3965-74. 17485350 10.2741/2363

[R4] Cohen CR, Gravelle L, Symekher S, Waiyaki P, Stamm WE, Kiehlbauch JA. Etiology of persistent tubo-ovarian abscess in Nairobi, Kenya. Infect Dis Obstet Gynecol. 2003;11(1):45-51. 12839632 10.1155/S1064744903000061 PMC1852266

[R5] Dimcic T, Gregoric M, Breznik V. Rapidly progressive infection of hand after a cat bite. Cureus. 2020 Jul 23;12(7):e9357. 32724758 10.7759/cureus.9357 PMC7381851

[R6] Dominy SS, Lynch C, Ermini F, Benedyk M, Marczyk A, Konradi A, Nguyen M, Haditsch U, Raha D, Griffin C, Holsinger LJ, Arastu-Kapur S, Kaba S, Lee A, Ryder MI, Potempa B, Mydel P, Hellvard A, Adamowicz K, Hasturk H, Walker GD, Reynolds EC, Faull RLM, Curtis MA, Dragunow M, Potempa J. Porphyromonas gingivalis in Alzheimer’s disease brains: Evidence for disease causation and treatment with small-molecule inhibitors. Sci Adv. 2019 Jan 23;5(1):eaau3333. 30746447 10.1126/sciadv.aau3333 PMC6357742

[R7] Fernandez-Canigia L, Cejas D, Gutkind G, Radice M. Detection and genetic characterization of β-lactamases in Prevotella intermedia and Prevotella nigrescens isolated from oral cavity infections and peritonsillar abscesses. Anaerobe. 2015 Jun; 33:8-13. 25623818 10.1016/j.anaerobe.2015.01.007

[R8] Gilpin DF, Nixon KA, Bull M, McGrath SJ, Sherrard L, Rolain JM, Mahenthiralingam E, Elborn JS, Tunney MM. Evidence of persistence of Prevotella spp. in the cystic fibrosis lung. J Med Microbiol. 2017 Jun; 66(6):825-32. 28604331 10.1099/jmm.0.000500

[R9] Golynska M, Polkowska I, Bartoszcze-Tomaszewska M, Sobczynska-Rak A, Matuszewski L. Molecular-level evaluation of selected periodontal pathogens from subgingival regions in canines and humans with periodontal disease. J Vet Sci. 2017 Mar 30;18(1):51-8. 27297417 10.4142/jvs.2017.18.1.51 PMC5366302

[R10] Gomes BP, Jacinto RC, Pinheiro ET, Sousa EL, Zaia AA, Ferraz CC, Souza-Filho FJ. Porphyromonas gingivalis, Porphyromonas endodontalis, Prevotella intermedia and Prevotella nigrescens in endodontic lesions detected by culture and by PCR. Oral Microbiol Immunol. 2005 Aug; 20(4):211-5. 15943764 10.1111/j.1399-302X.2005.00214.x

[R11] Hamada N, Takahashi Y, Watanabe K, Kumada H, Oishi Y, Umemoto T. Molecular and antigenic similarities of the fimbrial major components between Porphyromonas gulae and P. gingivalis. Vet Microbiol. 2008 Apr 1;128(1-2):108-17. 17977673 10.1016/j.vetmic.2007.09.014

[R12] Kolenbrander PE, Palmer RJ Jr, Periasamy S, Jakubovics NS. Oral multispecies biofilm development and the key role of cell-cell distance. Nat Rev Microbiol. 2010 Jul;8(7):471-80. 20514044 10.1038/nrmicro2381

[R13] Miceli DD, Zelarayan GS, Garcia JD, Fernandez V, Ferraris S. Diabetes mellitus remission in a cat with hyperadrenocorticism after cabergoline treatment. JFMS Open Rep. 2021 Jul 13;7(2):20551169211029896. 34345435 10.1177/20551169211029896 PMC8283091

[R14] Nomura R, Inaba H, Yasuda H, Shirai M, Kato Y, Murakami M, Iwashita N, Shirahata S, Yoshida S, Matayoshi S, Yasuda J, Arai N, Asai F, Matsumoto-Nakano M, Nakano K. Inhibition of Porphyromonas gulae and periodontal disease in dogs by a combination of clindamycin and interferon alpha. Sci Rep. 2020 Feb 20;10(1):3113. Erratum in: Sci Rep. 2020 Apr 24;10(1):7295. 32080231 10.1038/s41598-020-59730-9 PMC7033253

[R15] Nonaka S, Kadowaki T, Nakanishi H. Secreted gingipains from Porphyromonas gingivalis increase permeability in human cerebral microvascular endothelial cells through intracellular degradation of tight junction proteins. Neurochem Int. 2022 Mar;154:105282. 35032577 10.1016/j.neuint.2022.105282

[R16] O’Neill DG, Church DB, McGreevy PD, Thomson PC, Brodbelt DC. Prevalence of disorders recorded in cats attending primary-care veterinary practices in England. Vet J. 2014 Nov;202(2):286-91. 25178688 10.1016/j.tvjl.2014.08.004

[R17] Ozavci V, Erbas G, Parin U, Yuksel HT, Kirkan S. Molecular detection of feline and canine periodontal pathogens. Vet Anim Sci. 2019 Aug 27;8:100069. 32734086 10.1016/j.vas.2019.100069 PMC7386636

[R18] Rodrigues MX, Bicalho RC, Fiani N, Lima SF, Peralta S. The subgingival microbial community of feline periodontitis and gingivostomatitis: Characterization and comparison between diseased and healthy cats. Sci Rep. 2019 Aug 26;9(1):12340. 31451747 10.1038/s41598-019-48852-4 PMC6710259

[R19] Rothe K, Tsokos M, Handrick W. Animal and human bite wounds. Dtsch Arztebl Int. 2015 Jun 19;112(25):433-42. 26179017 10.3238/arztebl.2015.0433 PMC4558873

[R20] Sanai Y, Persson GR, Starr JR, Luis HS, Bernardo M, Leitao J, Roberts MC. Presence and antibiotic resistance of Porphyromonas gingivalis, Prevotella intermedia, and Prevotella nigrescens in children. J Clin Periodontol. 2002 Oct;29(10):929-34. 12445225 10.1034/j.1600-051x.2002.291008.x

[R21] Shenbakam P, Rao RJ, Prabhu S, Srirangarajan S, Rudresh V. Influence of antibacterial effects of tetracycline, laser, and photodynamic therapy on cell viability, cell damage, and virulence of Porphyromonas gingivalis. Photodiagnosis Photodyn Ther. 2021 Dec; 36:102617. 34740837 10.1016/j.pdpdt.2021.102617

[R22] Spears JK, Vester B, Gardner C, Li Q. Development of the oral microbiome in kittens. Companion Animal Nutrition (CAN) Summit: The Nexus of Pet and Human Nutrition: Focus on Cognition and Microbiome; Helsinki, Finland; 2017 4-7 May.

[R23] Takahashi N, Yamada T. Glucose metabolism by Prevotella intermedia and Prevotella nigrescens. Oral Microbiol Immunol. 2000 Jun;15(3):188-95. 11154402 10.1034/j.1399-302x.2000.150307.x

[R24] Tett A, Pasolli E, Masetti G, Ercolini D, Segata N. Prevotella diversity, niches and interactions with the human host. Nat Rev Microbiol. 2021 Sep;19(9):585-99. 34050328 10.1038/s41579-021-00559-y PMC11290707

[R25] Usui M, Onizuka S, Sato T, Kokabu S, Ariyoshi W, Nakashima K. Mechanism of alveolar bone destruction in periodontitis – Periodontal bacteria and inflammation. Jpn Dent Sci Rev. 2021 Nov;57:201-8. 34703508 10.1016/j.jdsr.2021.09.005 PMC8524191

[R26] Venkataraman A, Almas K. Rheumatoid arthritis and periodontal disease. An update. N Y State Dent J. 2015 Aug-Sep;81(5):30-6. 26521325

[R27] Wegner N, Wait R, Sroka A, Eick S, Nguyen KA, Lundberg K, Kinloch A, Culshaw S, Potempa J, Venables PJ. Peptidylarginine deiminase from Porphyromonas gingivalis citrullinates human fibrinogen and α-enolase: Implications for autoimmunity in rheumatoid arthritis. Arthritis Rheum. 2010 Sep;62(9):2662-72. 20506214 10.1002/art.27552 PMC2941529

[R28] Whyte A, Gracia A, Bonastre C, Tejedor MT, Whyte J, Monteagudo LV, Simon C. Oral disease and microbiota in free-roaming cats. Top Companion Anim Med. 2017 Sep;32(3):91-5. 29291775 10.1053/j.tcam.2017.07.003

